# Research progress on the treatment and nursing of sensorineural hearing loss

**DOI:** 10.3389/fnins.2023.1199946

**Published:** 2023-06-06

**Authors:** Fangfang Liu, Baoai Han, Xuhong Zhou, Shuo Huang, Jing Huang

**Affiliations:** Department of Otorhinolaryngology, Head and Neck Surgery, Zhongnan Hospital of Wuhan University, Wuhan, China

**Keywords:** sensorineural hearing loss, treatment, nursing, review, progress

## Abstract

This article provides a comprehensive review of the progress in the treatment and care of sensorineural hearing loss (SNHL), which is a common disease in the field of otolaryngology. In recent years, the incidence of SNHL has been on the rise due to factors such as fast-paced lifestyles, work pressure, and environmental noise pollution, which have a significant impact on the quality of life of patients. Therefore, the study of the treatment and care of SNHL remains a hot topic in the medical community. Despite significant advances in this field, there are still some challenges and limitations. For example, there is currently no single method that can completely cure SNHL, and the effectiveness of treatment may vary significantly among individuals. In addition, due to the complex etiology of SNHL, the prognosis of patients may vary greatly, requiring the development of personalized treatment plans and care strategies. To address these challenges, continuous research is needed to explore new treatment methods and care models to improve the quality of life of patients. In addition, there is a need for health education programs for the general public to raise awareness of SNHL and promote preventive measures to reduce its incidence. The ultimate goal is to ensure the sustainable development of the field of SNHL treatment and care, thus ensuring the health and well-being of affected individuals.

## Introduction

Sensorineural hearing loss (SNHL) is a common otolaryngologic disorder characterized by damage to the auditory nerve resulting in decreased or complete loss of hearing (Gregory et al., 2023; Matsunaga and Nakagawa, 2023; Zine and Fritzsch, 2023). The incidence of this disease has been increasing in recent years, which may be associated with factors such as fast-paced lifestyles, high-intensity work, and environmental noise pollution in modern society (Al-Azzawi and Stapleton, 2023; Saba et al., 2023). The treatment and care of this disease has been a hot topic in the medical field.

Although there have been many studies on the treatment and care of SNHL, there are still many questions about its pathogenesis and mechanisms. In many cases, the etiology of SNHL is not clear, especially in the absence of specific external stimuli and injuries (Marchioro et al., 2023). Therefore, research on its pathogenesis and mechanisms is still very necessary. From a molecular perspective, SNHL may involve a range of factors such as gene mutations, epigenetic changes, and protein abnormalities (Miguel et al., 2018; Jabbari Moghadam et al., 2023; Kelleci and Golebetmaz, 2023). Some gene mutations may lead to abnormal or disrupted function of the auditory nerve, leading to the occurrence of SNHL. In addition, epigenetic changes such as DNA methylation and histone modifications have also been found to be associated with SNHL, which may affect gene expression and function (Friedman and Avraham, 2009; Hou et al., 2016; Leso et al., 2020; Flook et al., 2021). Furthermore, protein abnormalities such as synaptic protein deficiency and metabolic enzyme dysfunction are also related to the occurrence of SNHL (Liu et al., 2022). In addition to the molecular level, the etiology and mechanisms of SNHL also involve various aspects such as neurodegenerative diseases, infection, poisoning, and hypoxia. For example, some neurodegenerative diseases such as presbycusis and acoustic neuroma may lead to SNHL (Paciello et al., 2023a, b). Infectious diseases such as rubella, measles, and influenza virus infections may also lead to SNHL (Moseley et al., 2023). Additionally, toxins such as ototoxic drugs and organic solvents may also cause damage to the auditory nerve, resulting in the occurrence of SNHL (Wang et al., 2023).

In terms of the etiology and mechanisms of SNHL, one of the research focuses is to search for potential therapeutic and preventive strategies. For example, treatment methods targeting certain gene mutations and epigenetic changes have been proposed in research. In addition, some studies have suggested that certain drugs and compounds may also have a positive impact on the development and progression of SNHL. For instance, drugs such as antioxidants, neurotrophins, and immunomodulators have been shown to play a role in protecting cochlear and auditory nerve function (Paciello et al., 2023a,b). Furthermore, recent studies have indicated that photoreceptors also play an important role in the occurrence and development of SNHL. Researchers have found that photosensitive proteins are expressed in the neurons and glial cells of the inner ear and are involved in the metabolism and photoresponse processes of inner ear cells. Therefore, the use of phototherapy to regulate inner ear cell metabolism and restore photoreceptor function has become a hot topic of research (Alkén et al., 2019).

In addition to finding treatment methods, preventing the occurrence of SNHL is also one of the research focuses. Although the protection of noise exposure and occupational hazards has received widespread attention, there are still many potential risk factors in areas such as home, entertainment, transportation, and military (Manickam et al., 2023). Therefore, raising public awareness and taking corresponding preventive measures are of great significance in preventing the occurrence of SNHL.

In summary, the etiology and pathogenesis of SNHL are very complex, involving multiple factors and mechanisms. Currently, the treatment and prevention of SNHL mainly rely on drug therapy, auditory rehabilitation, and individualized intervention methods. However, effective treatment and prevention of SNHL still face certain challenges and difficulties. Therefore, in-depth research on the etiology and pathogenesis of SNHL, exploring new therapeutic and preventive strategies, is of great significance in improving patients’ quality of life.

## Treatment method

Sensorineural hearing loss (SNHL) is a common auditory disorder that affects an increasing number of individuals. Currently, there are several treatment methods available for this condition. Drug therapy, including oral and intravenous methods, is one of the most commonly used treatments. Improvement can be achieved through the use of drugs that improve microcirculation, provide neuro-nutrition, and hormonal drugs (Erni et al., 2021). The efficacy of drug therapy can be enhanced through methods such as intratympanic injection.

In addition to drug therapy, traditional Chinese medicine (TCM) is also an effective treatment for SNHL (Fei et al., 2019). TCM treatment involves internal medicine, acupuncture, and ear acupressure therapy, which regulate the meridians and promote the exchange of substances between blood and the labyrinth, allowing for repair and regeneration of the inner ear and improvement of the patient’s hearing level. TCM treatment usually requires a certain amount of time to take effect. Furthermore, hyperbaric oxygen therapy is also a method for treating SNHL (Rhee et al., 2018; Joshua et al., 2022). Hyperbaric oxygen therapy uses a high-pressure oxygen environment to quickly increase the blood oxygen content, tension, and diffusion in the patient’s inner ear, repairing the cochlea and vestibular nerve fibers, and improving hypoxia and ischemia. Hyperbaric oxygen therapy should be performed in a specialized medical institution and under the guidance of a doctor. Recently, stem cell transplantation has also gradually been applied to the treatment of SNHL (Baumgartner et al., 2021). Stem cell transplantation can repair the cell types in the damaged area through the migration and differentiation of stem cells, and has the potential for therapeutic application. Although the efficacy of stem cell transplantation requires further research and validation, it has promising prospects for development.

In summary, treatment methods for SNHL include medication, traditional Chinese medicine, hyperbaric oxygen therapy, and stem cell transplantation, each with unique mechanisms of action and indications. However, these treatments also have limitations and risks. Although medication is one of the main treatments for SNHL, its effectiveness is not always ideal. For example, some patients may not tolerate certain medication side effects, or certain drugs may cause serious adverse reactions. In addition, medication therapy requires long-term use to achieve better efficacy, which may cause psychological and economic burdens for some patients. Although traditional Chinese medicine is an effective treatment method, its efficacy requires accumulation over time, and there is individual variability. Moreover, experienced physicians are needed to ensure the safety and effectiveness of treatment. The mechanism of hyperbaric oxygen therapy is to improve the hypoxic state of the inner ear by increasing the oxygen content of blood. Although this method can bring significant benefits in some cases, it requires treatment in a hyperbaric oxygen chamber, and there may be some discomfort during treatment, such as headaches and dizziness. Stem cell transplantation therapy is an emerging treatment method, and its efficacy needs to be further confirmed by research. Stem cell transplantation therapy also carries some risks and uncertainties, such as potential immune reactions and abnormal cell proliferation. In addition, treatment methods targeting certain gene mutations and epigenetic changes have been proposed in research, which provides new ideas for the treatment of SNHL. Therefore, the treatment of SNHL needs to take into account factors such as the patient’s physical condition, the severity of the disease, treatment goals, and risks, and adopt an individualized treatment plan (Löfvenberg et al., 2022). At the same time, healthcare professionals need to establish good communication and trust with patients, help them solve problems during treatment, and improve patient compliance and treatment efficacy.

## Nursing method

For patients with sensorineural hearing loss (SNHL), nursing care should include psychological, dietary, lifestyle, medication, hyperbaric oxygen, and traditional Chinese massage interventions. The specific methods are as follows:

### Psychological care

Patients with SNHL often experience dizziness, ear fullness, restlessness, anxiety, and tension (Job et al., 2023). Therefore, it is important to maintain a quiet environment in the hospital room, avoid noise, and arrange single rooms whenever possible. For those who experience dizziness, they should be advised to rest in bed. Communication between the caregiver and the patient should be strengthened to understand the patient’s psychological status, provide care, support, and encouragement, and help the patient maintain a positive attitude and cooperate with treatment. At the same time, the caregiver should evaluate the patient’s role behavior, classify the patient’s role transformation and adaptation into five aspects: lack, conflict, decline, reinforcement, and abnormality, and provide personalized nursing interventions to help the patient adapt to the changes as soon as possible (Chandrasekhar et al., 2019). Nurses can provide patients with disease knowledge education, patiently answer questions, improve the patient’s understanding of the disease, establish a good nurse–patient relationship, and improve the patient’s treatment compliance and cooperation. Patients should be provided with psychological care and counseling to strengthen their psychological defenses, help relieve negative emotions, and use entertainment activities to divert patients’ attention from the disease and relieve their stress response to maintain the best physical and mental state to cooperate with treatment.

### Dietary care

During the treatment period, patients should eat low-salt, low-fat, and light foods. They should eat foods containing vitamin E, vegetables, and fresh fruits, such as egg yolk, vegetable oil, beans, and so on. Patients should quit smoking because nicotine in cigarettes can cause vascular spasm, damage endothelial cells, cause platelet adhesion, aggregation, and formation of thrombosis, which affects treatment efficacy. Patients can drink a small amount of alcohol to promote blood circulation and metabolism, and it is recommended to drink light tea because tea contains a variety of vitamins, amino acids, and proteins (Kociszewska et al., 2021).

### Life care

Patients with SNHL should be provided with a quiet and comfortable treatment environment to avoid excessive noise. Patients should avoid using headphones or making long phone calls to prevent exacerbation of hearing damage. During treatment, patients should maintain emotional stability and engage in relaxing activities such as listening to music or reading to avoid prolonged phone or television use. Patients should also maintain a regular sleep schedule, prioritize sufficient sleep, and create a relaxing sleep environment. Daily, patients should aim for a minimum of 7 h of restful sleep. Additionally, appropriate exercise should be encouraged to reduce thrombosis, prevent hyperlipidemia, and minimize the impact of slow blood flow on inner ear microcirculation.

### Medication care

During treatment with corticosteroids, patients’ eating and sleeping patterns should be monitored (Plontke et al., 2022). If there is a noticeable increase in appetite, patients should be advised to control their diet. Many patients also experience sleep disorders, and patients should be informed of the drug’s adverse effects and encouraged to minimize daytime sleep to improve nighttime sleep quality. Inhibiting red blood cell and platelet aggregation, lowering blood viscosity, and dissolving blood clots are the goals of batroxobin drugs, which can also increase vascular permeability, improve blood supply, and improve hearing (Wang et al., 2021). However, batroxobin may sometimes cause complications such as increased eosinophils, leukocytosis or leukopenia, decreased red blood cells, and hemoglobin. Digestive symptoms such as nausea, vomiting, stomach pain, and loss of appetite may also occur. In terms of mental and neurological effects, patients may experience dizziness, headache, unsteady gait, or numbness. Therefore, close observation of the patient’s condition is essential, and drug administration should be discontinued in case of abnormal reactions. Meanwhile, patients should be monitored for signs of bleeding or other adverse events, and preventative measures should be taken. During medication treatment for SNHL, nurses should increase surveillance and closely monitor changes in patients’ condition, vital signs, and blood counts. Any abnormalities should be promptly reported to the physician.

### Nursing care for hyperbaric oxygen therapy

When performing hyperbaric oxygen therapy, attention should be paid to the following (Olex-Zarychta, 2020; Ahn et al., 2021; Joshua et al., 2022): (1) Before entering the chamber, analyze the patient’s Eustachian tube function and check the patency of the Eustachian tube. For patients with poor Eustachian tube function or allergic rhinitis, nasal spray medication should be used to contract the nasal mucosa and relieve symptoms; instruct patients to chew gum or eat fruit during pressurization to maintain Eustachian tube patency. (2) Due to the airtight environment of the oxygen chamber, it may cause anxiety and worsen depressive symptoms in patients. Individualized psychological counseling should be provided to the patient, paying attention to changes in their psychological state, giving active care and comfort to relieve negative emotions. Introduce the treatment method and expected effects to improve their treatment compliance. Patients may also be shown the treatment environment in advance, and peer education and sharing experiences can be used. (3) During oxygen chamber treatment, maintain the temperature in the chamber, with temperatures ranging from 24°C to 28°C in summer and 18°C to 22°C in winter. Help patients adjust their comfortable position, play soothing music to ease their nervousness, instruct them on the prevention of common complications, strictly control treatment time and oxygen inhalation time, adjust the oxygen inhalation method, and allow for 30 min of rest inside the chamber after 30 min of oxygen inhalation. The oxygen mask should be in close contact with the face to avoid inhaling mixed air, which may affect the therapeutic effect and cause oxygen poisoning. Instruct patients to breathe independently, and prohibit breath-holding and coughing movements to prevent lung barotrauma. If a patient experiences coughing, chest pain, or restlessness, immediate intervention is required. (4) Before leaving the chamber, control the decompression speed and rate, observe whether the patient has any vomiting, breathing disorders, or restlessness, and stop immediately if abnormalities are found to prevent decompression sickness.

### Guidance for traditional Chinese medicine massage

Traditional Chinese medicine massage involves kneading, pushing, pulling, and pulling the earlobe. Kneading the temple and other methods can be performed once a day for 20 min, with a 14-day course, to help improve ear activity, promote blood circulation in the ear, enhance ear blood supply function, and aid in disease recovery. Before the massage, explain the procedure to the patient and obtain their cooperation. Evaluate the patient’s skin condition at the massage site, checking for abrasions, nodules, and rashes. The massage pressure should be based on the patient’s tolerance, and stopped immediately if any discomfort is experienced.

## Discussion

**Figure 1:**
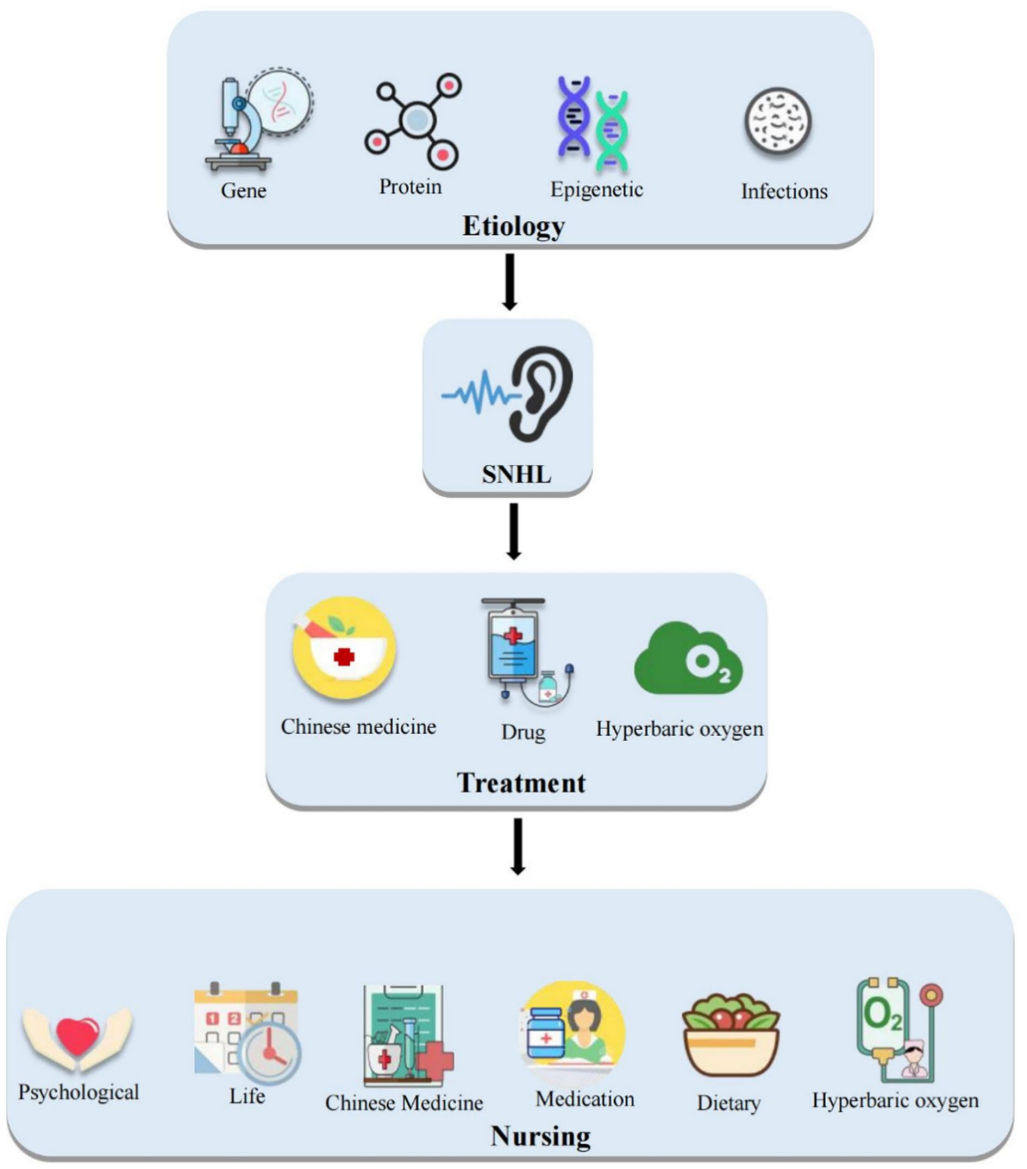
Schematic diagram of treatment and nursing research on sensorineural hearing loss.

In conclusion, significant progress has been made in the clinical treatment of SNHL (Figure 1). Therefore, high patient cooperation is required during the treatment process, and nurses should focus on patient-centered care, develop a planned and purposeful nursing plan, provide personalized care based on the patient’s condition, perform predictive assessments of potential risk factors, and collaborate with disease and medication observations to improve patient treatment compliance, and ultimately enhance treatment outcomes and hearing levels. Moreover, nurses should assist patients in building a positive physical and mental state and correct understanding of their disease to alleviate anxiety and tension and improve treatment compliance to achieve the goal of improving treatment outcomes.

Additionally, SNHL prevention is crucial, and nurses should focus on disease prevention education during the nursing process, such as maintaining healthy lifestyles and avoiding noisy environments to reduce the risk of illness (Rahimi et al., 2023). The development of emerging therapies, new technologies, and ongoing clinical trials has promoted the advancement of treatment and care for SNHL, resulting in improved prognosis. A multidisciplinary approach involving otolaryngologists, audiologists, speech pathologists, and other healthcare professionals has provided optimal guidance for the treatment and care of SNHL patients (Copeland and Pillsbury 3rd., 2004; Gay et al., 2022).

In summary, the treatment and care of SNHL are comprehensive processes that require the joint efforts of medical staff and active patient cooperation. Guided by patient-centered care philosophy, nurses should develop personalized treatment plans based on the patient’s actual condition, provide comprehensive management and observation during the treatment process to enhance treatment outcomes and improve patients’ quality of life.

## Author contributions

FL and BH: conception and design. FL and JH: development of methodology. XZ and SH: analysis and interpretation of data. JH: writing, review, and/or revision of the manuscript. SH: study supervision. All authors contributed to the article and approved the submitted version.

## Conflict of interest

The authors declare that the research was conducted in the absence of any commercial or financial relationships that could be construed as a potential conflict of interest.

## Publisher’s note

All claims expressed in this article are solely those of the authors and do not necessarily represent those of their affiliated organizations, or those of the publisher, the editors and the reviewers. Any product that may be evaluated in this article, or claim that may be made by its manufacturer, is not guaranteed or endorsed by the publisher.
